# Supplementary material for: In search of diverse and connected teams: A computational approach to assemble diverse teams based on members’ social networks
Source: PLoS One. 2022 Nov 9;17(11):e0276061. doi: 10.1371/journal.pone.0276061 (PMC9645621; doi:10.1371/journal.pone.0276061)
Supplement: S1 File — S1 Fig: Simulations using the Diameter metric. S2 Fig: Simulations using the Minimum Spanning Tree (MST) metric. S1 Table: Diameter Case. S2 Table: Minimum Spanning Tree Case. S3 Table: Team combinations’ average proportion of hops. (PDF) [file pone.0276061.s001.pdf]

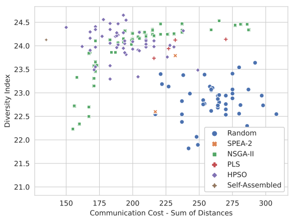

(a) MyDreamTeam: Experiment A

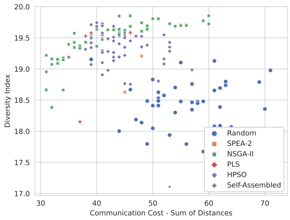

(b) MyDreamTeam: Experiment B

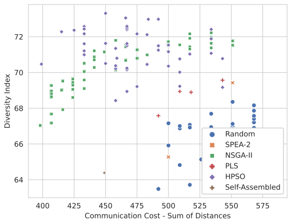

(c) MyDreamTeam: Experiment C

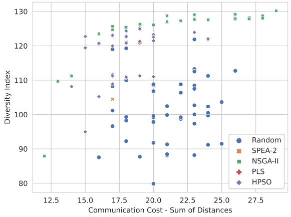

(d) Bibsonomy: Nature papers

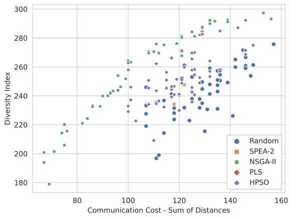

(e) Bibsonomy: Science papers

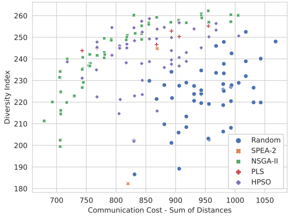

(f) Bibsonomy: Physica A. papers

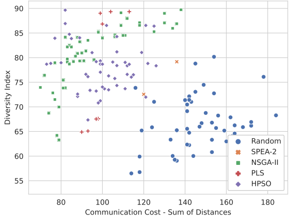

(g) GHTorrent: Python repositories

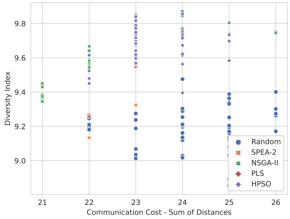

(h) GHTorrent: Java repositories

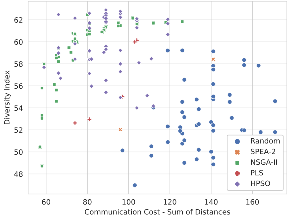

(i) GHTorrent: Ruby repositories

Figure 1: **Simulations using the Diameter metric.** Results of NSGA-II, SPEA-2, PLS, HPSO, and Random assignment. For each team, we created a network using the prior collaborations and calculated the diameter of each connected component. For isolated members, we considered the diameter of the collaboration network. We sum all diameters for each team.

	Hypervolume					Unique Non-dominated Front Ratio				
	HPSO	NSGA-II	PLS	Random	SPEA-2	HPSO	NSGA-II	PLS	Random	SPEA-2
MDT Case A	**45,595**	45,219	43,056	42,129	42,402	**1.00**	0	0	0	0
MDT Case B	39,003	**39,106**	38,437	37,559	37,548	0.43	**0.57**	0	0	0
MDT Case C	**117,294**	115,522	104,855	102,987	103,925	**0.83**	0.17	0	0	0
Bibsonomy Nature	374,000	**388,878**	360,433	363,584	361,032	0.29	**0.71**	0	0	0
Bibsonomy Science	831,666	**869,030**	822,415	796,910	713,555	0	**1.00**	0	0	0
Bibsonomy Physics A	588,598	**604,864**	574,246	544,136	530,560	0.29	**0.64**	0.07	0	0
GHTorrent Case Python	172,689	**172,864**	170,421	150,964	148,734	**0.67**	0.33	0	0	0
GHTorrent Case Java	19,495	**19,533**	18,884	18,743	18,442	**0.67**	0.33	0	0	0
GHTorrent Case Ruby	**122,045**	121,296	115,659	112,536	110,955	**0.50**	**0.50**	0	0	0

Table 1: **Diameter Case**: Hypervolume and Unique Non-dominated Front Ratio values for the five methods across the datasets. The best results are marked in bold.

	Hypervolume					Unique Non-dominated Front Ratio				
	HPSO	NSGA-II	PLS	Random	SPEA-2	HPSO	NSGA-II	PLS	Random	SPEA-2
MDT Case A	44,700	**45,181**	42,882	41,521	42,144	0.36	**0.64**	0	0	0
MDT Case B	**38,953**	38,598	37,429	36,976	36,213	**0.67**	0.33	0	0	0
MDT Case C	113,254	**115,613**	108,107	103,754	102,122	**0.73**	0.27	0	0	0
Bibsonomy Nature	375,778	**393,705**	370,648	376,121	369,246	0.39	**0.61**	0	0	0
Bibsonomy Science	813,414	**868,466**	795,042	803,358	777,496	0	**1.00**	0	0	0
Bibsonomy Physics A	591,891	**618,213**	543,217	543,410	507,329	0.19	**0.81**	0	0	0
GHTorrent Case Python	174,551	**174,601**	168,046	153,444	123,726	**0.57**	0.43	0	0	0
GHTorrent Case Java	**18,967**	18,787	18,397	18,284	18,089	**0.86**	0.14	0	0	0
GHTorrent Case Ruby	118,832	**122,320**	116,540	108,744	112,680	0.38	**0.63**	0	0	0

Table 2: **Minimum Spanning Tree Case**: Hypervolume and Unique Non-dominated Front Ratio values for the five methods across the datasets. The best results are marked in bold.

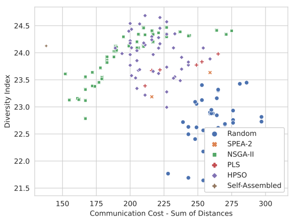

(a) MyDreamTeam: Experiment A

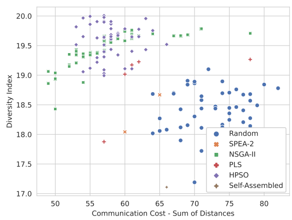

(b) MyDreamTeam: Experiment B

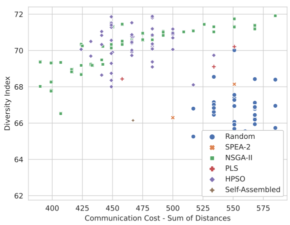

(c) MyDreamTeam: Experiment C

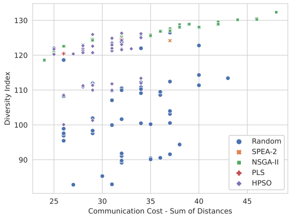

(d) Bibsonomy: Nature papers

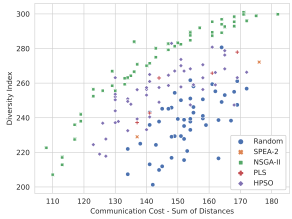

(e) Bibsonomy: Science papers

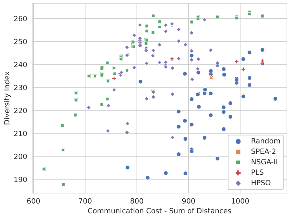

(f) Bibsonomy: Physica A. papers

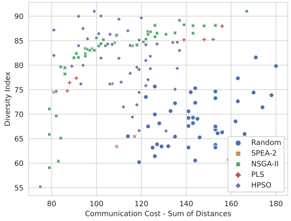

(g) GHTorrent: Python repositories

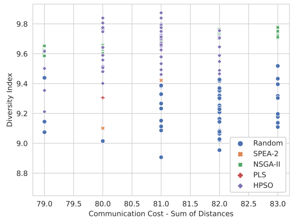

(h) GHTorrent: Java repositories

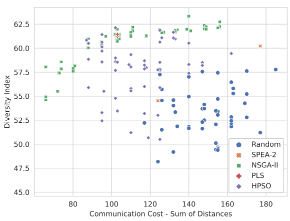

(i) GHTorrent: Ruby repositories

Figure 2: **Simulations using the Minimum Spanning Tree (MST) metric.** Results of NSGA-II, SPEA-2, PLS, HPSO, and Random assignment. For each team, we created a network using the prior collaborations and calculated the MST of each connected component. The cost of a spanning tree is simply the sum of its edges. For isolated members, we sum the diameter of the collaboration network.

Database	Algorithm	0	1	2	3	4	5	6	7	8	9	10	11	12	13
Bibsonomy (Nature)	HPSO	0.20	**0.49**	0.33	0.14										
Bibsonomy (Nature)	NSGA-II	0.20	**0.60**	0.30	0.12										
Bibsonomy (Nature)	PLS	0.20	**0.46**	0.35	0.16										
Bibsonomy (Nature)	Random	0.20	**0.40**	0.38	0.17										
Bibsonomy (Nature)	SPEA-2	0.20	**0.47**	0.34	0.09										
Bibsonomy (Physica)	HPSO	**0.24**	0.04	0.16	0.15	0.15	0.17	0.15	0.13	0.11	0.11	0.10	0.09	0.15	0.11
Bibsonomy (Physica)	NSGA-II	**0.24**	0.03	0.16	0.15	0.16	0.16	0.15	0.13	0.11	0.12	0.11	0.09	0.09	0.08
Bibsonomy (Physica)	PLS	**0.24**	0.03	0.17	0.15	0.15	0.17	0.15	0.13	0.11	0.11	0.13	0.09	0.08	
Bibsonomy (Physica)	Random	**0.24**	0.03	0.13	0.13	0.15	0.16	0.15	0.13	0.12	0.12	0.11	0.10	0.11	0.09
Bibsonomy (Physica)	SPEA-2	**0.24**	0.03	0.13	0.14	0.15	0.15	0.16	0.13	0.14	0.11	0.10	0.08	0.10	
Bibsonomy (Science)	HPSO	0.20	0.37	**0.40**	0.32	0.08									
Bibsonomy (Science)	NSGA-II	0.20	**0.39**	0.39	0.32	0.21									
Bibsonomy (Science)	PLS	0.20	**0.38**	0.38	0.32	0.08									
Bibsonomy (Science)	Random	0.20	0.36	**0.40**	0.32	0.09									
Bibsonomy (Science)	SPEA-2	0.20	**0.40**	0.39	0.35	0.16									
GHTorrent (Java)	HPSO	0.20	**0.77**	0.24											
GHTorrent (Java)	NSGA-II	0.20	**0.78**	0.24											
GHTorrent (Java)	PLS	0.20	**0.76**	0.28											
GHTorrent (Java)	Random	0.20	**0.76**	0.30											
GHTorrent (Java)	SPEA-2	0.20	**0.76**	0.26											
GHTorrent (Python)	HPSO	0.23	0.23	0.26	**0.32**	0.17	0.08								
GHTorrent (Python)	NSGA-II	0.23	0.23	**0.30**	0.29	0.14	0.08								
GHTorrent (Python)	PLS	0.22	0.22	**0.38**	0.26	0.18	0.13								
GHTorrent (Python)	Random	0.23	0.15	0.29	**0.34**	0.17	0.08								
GHTorrent (Python)	SPEA-2	0.23	0.15	**0.32**	0.30	0.16	0.08								
GHTorrent (Ruby)	HPSO	0.20	**0.38**	0.29	0.22	0.20	0.14	0.08	0.10	0.08					
GHTorrent (Ruby)	NSGA-II	0.20	**0.42**	0.28	0.17	0.19	0.13	0.12	0.08						
GHTorrent (Ruby)	PLS	0.20	**0.37**	0.25	0.22	0.21	0.16	0.08	0.08	0.08					
GHTorrent (Ruby)	Random	0.20	**0.34**	0.27	0.21	0.21	0.14	0.09	0.09	0.08					
GHTorrent (Ruby)	SPEA-2	0.20	**0.31**	0.29	0.21	0.21	0.14	0.10							
MDT Project Case A	HPSO	**0.47**	0.08	0.22	0.23	0.18	0.13	0.09							
MDT Project Case A	NSGA-II	**0.50**	0.08	0.21	0.21	0.15	0.13	0.10							
MDT Project Case A	PLS	**0.49**	0.07	0.25	0.16	0.14	0.15	0.14							
MDT Project Case A	Random	**0.53**	0.05	0.17	0.18	0.14	0.14	0.10							
MDT Project Case A	SPEA-2	**0.48**	0.05	0.21	0.24	0.13	0.13	0.11							
MDT Project Case B	HPSO	0.24	0.27	**0.54**											
MDT Project Case B	NSGA-II	0.24	0.25	**0.56**											
MDT Project Case B	PLS	0.24	0.24	**0.57**											
MDT Project Case B	Random	0.24	0.20	**0.62**											
MDT Project Case C	HPSO	**0.64**	0.03	0.27	0.26	0.24	0.24	0.24	0.24	0.23	0.22				
MDT Project Case C	NSGA-II	**0.62**	0.04	0.28	0.26	0.27	0.27	0.26	0.28	0.25	0.22				
MDT Project Case C	PLS	**0.61**	0.05	0.22	0.29	0.31	0.26	0.22	0.22						
MDT Project Case C	Random	**0.66**	0.02	0.25	0.27	0.27	0.26	0.26	0.25	0.25	0.22				
MDT Project Case C	SPEA-2	**0.63**	0	0.29	0.27	0.28	0.22	0.28	0.22	0.22					

Table 3: **Team combinations' average proportion of hops.** This table shows the average proportion of hops between team members. The average was calculated by team. We aggregated teams' number of hops based on the 50 solutions in each population, then by algorithms, and finally by datasets. The header shows the number of hops size n. When team members did not have any prior relationships, we counted them as hop 0. Members with prior relationships are separated by one hop. Since these proportion numbers are averages, they do not sum 100%.
