# Supplementary material for: In search of diverse and connected teams: A computational approach to assemble diverse teams based on members’ social networks
Source: PLoS One. 2022 Nov 9;17(11):e0276061. doi: 10.1371/journal.pone.0276061 (PMC9645621; doi:10.1371/journal.pone.0276061)
Supplement: Attachment — Submitted filename: Response to Reviewers.pdf [file pone.0276061.s003.pdf]

September 9th, 2022

Dear Dr. Lozano and reviewers,

We thank you and appreciate your precious time and efforts in revising our manuscript. Your valuable and insightful observations allowed us to improve this article. We are pleased to submit our revised paper to address the most recent comments.

R1 requested providing more intuition about the metrics used to evaluate the algorithms. We added more context and rationale in the Metrics sub-section and Results section. Also, we have fixed the typos identified by R1. In addition, two authors have proofread this new version and fixed typos and grammar errors.

We also want to thank R3 for the kind comments and for taking the time to revise this resubmission.

In the remainder of this document, we address the points raised in the last review and explain the steps we have taken to address these concerns in this revised manuscript.

We appreciate the opportunity to revise and resubmit our paper, and we sincerely hope we can share our contribution at PLOS One. Thank you.

Sincerely,
The Authors

Editor's meta-review

As you can see below, both reviewers have recommended publication. However, Reviewer #1 has asked for some intuition on the quality metrics introduced, and has pointed out some minor issues. Please, address them in your revised version.

In this revised version, we elaborate on the rationale of the metrics used in this study. More details are provided in response to R1 in this letter.

Moreover, since PLOS ONE does not proof-read manuscript, I suggest revising the text for small typos and shortcomings. For instance, the word 'otherwise' is written twice in line 979.

Two authors have proofread this new version and fixed typos and grammar errors.

Reviewer 1

One request I have is for the authors is to provide intuition about the metrics that they have used for evaluating the NSGA-II algorithm and why they matter in the specific context of team formation. For example, intuitively, why is a higher hypervolume value preferred and what qualities about a selection of teams does it demonstrate?

In this revised version, we provide more insight into the metrics used by this study. In summary, higher hypervolume scores show that teams with higher levels of diversity and familiarity can be found. The Unique Non-dominated Front Ratio shows how many high-diversity/high-familiarity team combinations were found by only one algorithm. Lastly, evaluating the running time is essential since some problems will require large participant pools to be assigned to teams. We develop the rationale and intuition of these metrics in the Metrics sub-section and Results section.

Line 318-319 is awkwardly phrased and I had a hard time reading it at first. The clause "and it has been classified as an NP-hard problem" in effect refers to the minimization problem but with the sentence it is conjoined with, it seems as if it refers to computing the communication cost which in fact, can be done in polynomial time.

Thank you for pointing out this error. It was in fact referring to the minimization problem and not to computing the communication cost. We removed the sentence and the resulting paragraph is the following:

"The goal is to minimize the average sum of shortest path lengths across all assembled teams in the individuals' network. Computing the sum of communication costs of a set of teams runs in $O(n^2)$ time."

Line 296: P_j & P_j => P_i & P_j

Fixed.
